# Pancytopenia Related to Splenic Angiosarcoma: A Case Report and Literature Review

**DOI:** 10.3390/hematolrep16040063

**Published:** 2024-10-18

**Authors:** Jakub Misiak, Bernard Sokołowski, Norbert Skrobisz, Mateusz Matczak, Marcin Braun

**Affiliations:** Department of Pathology, Chair of Oncology, Medical University of Lodz, 92-213 Lodz, Poland; misiakj98@gmail.com (J.M.); bernard.sokolowski@outlook.com (B.S.); norbert3535@icloud.com (N.S.); matczak.mateusz@icloud.com (M.M.)

**Keywords:** splenic angiosarcoma, bone marrow metastases, pancytopenia, myelodysplastic syndrome

## Abstract

Background: Angiosarcomas are highly aggressive malignancies with endothelial differentiation, presenting considerable challenges in oncology, especially when arising in rare locations such as the spleen. These tumors predominantly affect adults and are commonly found in the skin, breast, liver, or soft tissues, with more unusual occurrences in other organs. Angiosarcomas have a high propensity for metastasis, typically spreading to the liver, lungs, lymph nodes, and gastrointestinal tract. Splenic angiosarcoma, with fewer than 300 documented cases, is an especially rare and complex form of this malignancy. Case presentation: This report details a case of splenic angiosarcoma in a 45-year-old male, where bone marrow metastases were the first clinical presentation, initially mimicking myelodysplastic syndrome (MDS) due to persistent pancytopenia. Conclusions: The eventual identification of the splenic origin underscores the diagnostic difficulties and clinical challenges inherent in managing such atypical and rare presentations.

## 1. Introduction

Angiosarcoma is a malignant neoplasm with morphological or immunophenotypic features of endothelial differentiation. These aggressive tumors are generally found in adults, mainly in the skin, soft tissue, breast, liver, and, infrequently, in other organs such as the spleen [1]. To date, there are fewer than 300 reports of splenic angiosarcoma available in the literature [2] (Table 1). Angiosarcoma typically metastasizes to the liver, lungs, lymph nodes or bone [1,3,4]. Here, we present a rare case of angiosarcoma in the form of splenic angiosarcoma with bone marrow metastasis mimicking myelodysplastic syndrome.

## 2. Case Presentation

A 45-old-year male patient was admitted to the Hematology Department with a suspicion of myelodysplastic syndrome (MDS). He presented with petechiae, and blood tests showed a decreased hemoglobin level of 9.5 g/dL and thrombocytopenia at 24 k/mcL (2.4 × 10^4^/μL) (Table 2). Examination of *FLT3* and *NMP1* mutations was performed, but no abnormalities were detected. The presence of schistocytes in the peripheral blood smear, increasing liver enzyme levels, and the patient’s deteriorating condition were suggestive of acquired thrombotic thrombocytopenic purpura (aTTP). After a suspicion of aTTP, the following tests were performed: reticulocytes—0.110 × 10^6^/μL, INR—1.11, PT—12.8 s, APTT—23.8 s, LDH—978 U/L, haptoglobin—<0.1 g/L, total bilirubin—1.08 mg/dL, fibrinogen—188 mg/dL, and ADAMTS13 antibodies in the plasma, but they were not detected. Vitamin B12 and folic acid deficiencies were also excluded (1306 pg/mL and 6.15 ng/mL, respectively). The patient also experienced abdominal pain and periodic fever during hospitalization. The patient underwent three cycles of plasma exchange. However, due to the lack of improvement in blood morphology tests, a decision to discontinue the procedure was made.

**Figure 1:**
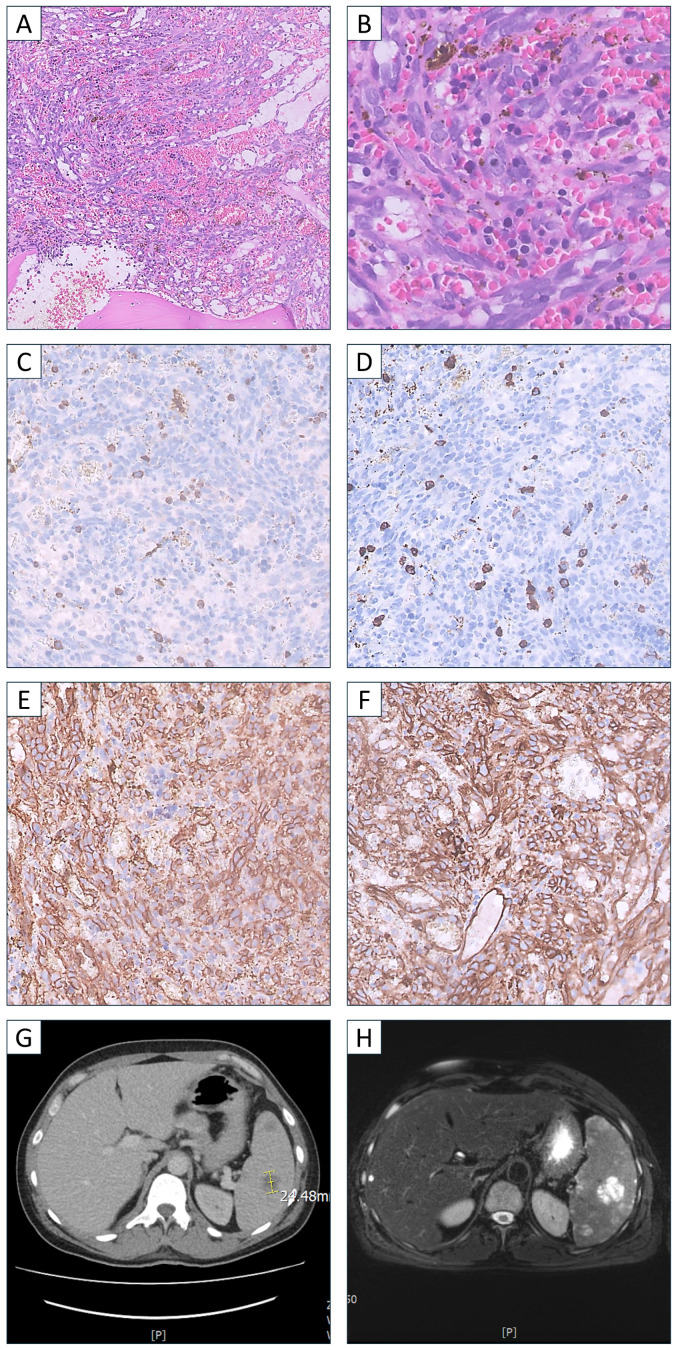
Histopathology examination diffuse infiltration of bone marrow by neoplastic cells. (**A**) Hematoxylin and eosin, 1× magnification; (**B**) Hematoxylin and eosin, 40× magnification. Immunohistochemical profiling of the neoplastic cells (**C**) MPO negative, 20× magnification; (**D**) CD15 negative, magnification 20×; (**E**) CD34 positive, 20×magnification; (**F**) CD31 positive, 20× magnification. A tumor lesion in a spleen in (**G**) computed tomography and (**H**) magnetic resonance imaging. The lesion in the central part of the spleen imitates cavernous angiomas. The heterogeneity of the lesions and the presence of hemorrhagic focus suggest a more aggressive clinical picture like angiosarcoma.

Due to the occurrence of a dry tap during bone marrow aspiration, a trephine biopsy was performed. The histopathological examination showed widespread infiltration of the bone marrow by elongated and epithelioid neoplastic cells. These cells created numerous pseudovascular spaces. Additionally, reticular fibrosis of grade 3 was present (Figure 1A,B). Immunohistochemical profiling of the neoplastic cells revealed positivity for vascular markers (CD34, CD31, CD117, SMA), and negativity for cytokeratins, mastocytic, myeloid, and lymphoid markers (CK AE1/AE3, MPO, CD15, CD61, CD71, CD123, CD138, CD20, CD3, TdT, CD25, MCT) (Figure 1C–F).

Whole-body contrast-enhanced computed tomography (CT) revealed numerous hypodense foci exclusively in the spleen (Figure 1G). Magnetic resonance imaging (MRI) also identified the lesions, with the largest measuring 36 × 21 mm and 32 × 30 mm, containing hemorrhagic deposits (Figure 1H). All the above imaging tests, including of the skeletal system, revealed no signs of metastatic remodeling. Thus, the final diagnosis was splenic angiosarcoma metastatic to bone marrow.

The patient underwent palliative treatment with paclitaxel at a dose of 60 mg/m^2^ every 7 days with prophylaxis of febrile neutropenia using short-acting G-CSF starting in February 2022. Radiotherapy targeting the primary tumor in the spleen was added in July 2022. After receiving a total of 29 cycles of paclitaxel without an effective response, a weekly infusion of doxorubicin at a dose of 7.5 mg/m^2^ was recommended, with dose reduction due to the patient’s general condition. Nine months after the beginning of treatment, the patient died.

## 3. Discussion

In the presented case, a diagnosis of bone marrow metastatic angiosarcoma originating from the spleen is discussed, highlighting a distinctive clinico-pathological scenario due to the myelodysplastic syndrome-like symptoms induced by the bone marrow metastases. The uniqueness of this case is underscored by the rare occurrence of such metastases, emphasizing the biological complexity and variability in the clinical presentation of angiosarcoma [28].

A particularly rare manifestation in the patient we described was bone metastases located between the trabeculae, directly infiltrating the bone marrow and impacting blood count results. The thrombocytopenia and petechiae observed in the patient initially suggested a hematopoietic disorder, such as myelodysplastic syndrome (MDS), leukemia, or thrombocytopenic purpura. The rarity of this manifestation contributed to the delay in reaching the final diagnosis.

In our patient’s case, typical imaging tests and blood tests were performed for the diagnosis of angiosarcoma. Both MRI and CT imaging are standard diagnostic tools for these tumors. However, the biopsy in this case differed from the usual approach. Typically, for solid tumors, a biopsy of the primary lesion or a lymph node affected by metastasis is performed. In our case, the patient underwent a procedure more common for hematopoietic tumors, namely a trephine biopsy. This biopsy allowed for the collection of metastatic tumor cells, which were subsequently characterized using immunohistochemistry (IHC). The results of the imaging tests and IHC together confirmed the final diagnosis of angiosarcoma of splenic origin.

Upon review of the literature (Table 1), it is evident that splenic angiosarcoma can manifest across a wide age range. For instance, a case involving a 25-year-old woman demonstrates that younger populations can also be affected by this disease [5,16,23]. Most reported instances involve symptoms of abdominal discomfort, pain, or palpable masses in the left epigastrium, which can serve as early indicators of the disease [5,6,7,20,23,24,27]. Additionally, while the liver is the most frequent site for metastases of splenic angiosarcoma, occurrences have been documented in bones and bone marrow (as in the presented case), peritoneum, lungs, kidneys, and potentially the thyroid gland, illustrating the extensive metastatic potential of angiosarcoma [25,26,27].

**Figure 2:**
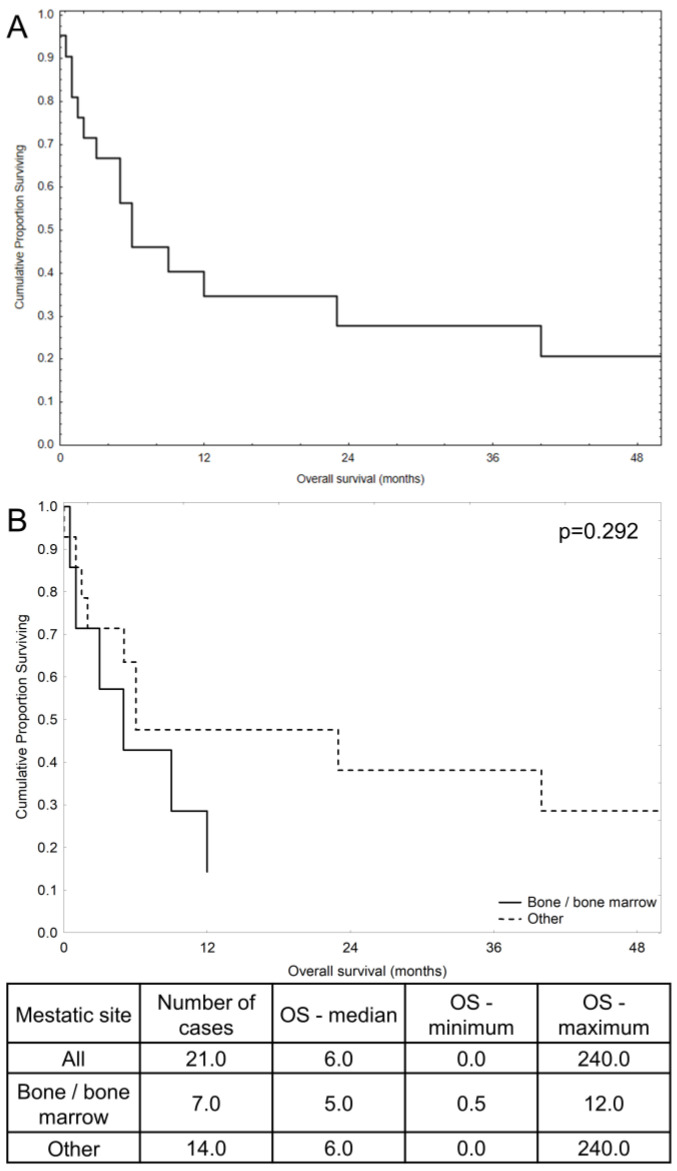
Kaplan–Meier curves along with the table with median and ranges for overall survival (OS) in months based on the available OS data presented in Table 1. (**A**) OS of all 21 patients reported in Table 1 and this case. (**B**) Comparison of OS between patients with bone/bone marrow metastases and other metastatic sites.

Current treatment approaches predominantly involve splenectomy followed by chemotherapy with agents such as paclitaxel [17,18], but the outcomes vary across patients with various presentations of the disease (Table 1, Figure 2). The presence of unique metastatic profiles and clinical presentations, such as in the bone marrow metastatic angiosarcoma case discussed here, accentuates the need for individualized treatment plans and further research into tailored therapeutic interventions. Notably, in cases with bone marrow metastases, regardless of patient age, survival rates are poor [8,23,26,27] (Figure 2). Our patient died within 9 months of treatment initiation, which is close to the median survival reported in the literature (Figure 2). This suggests that metastases to the bone marrow might be a significant prognostic factor, warranting further investigation into survival differences compared to other metastatic sites. An important aspect of both treatment and diagnostics in this case appears to be splenectomy. In the patient described, the disease primarily manifested as abnormalities in blood morphology tests, which were secondary to bone marrow metastases. These metastases led to the observed blood abnormalities. In cases of metastatic disease, surgery does not improve prognosis and additionally exposes the patient to potential complications. In this case, surgery was not feasible, not only due to the widespread nature of the disease, but also because of the blood abnormalities directly caused by bone marrow metastases. In summary, the use of palliative chemotherapy was the only viable option with a chance to prolong the patient’s life and slow the progression of the neoplastic disease.

Improving outcomes in this area remains a challenge for clinicians. In patients presenting with similar symptoms, it is worth considering immediate imaging tests to identify the primary tumor site. This approach enables the simultaneous diagnosis of both hematopoietic disorders and the potential development of a neoplastic process in other organs.

## 4. Conclusions

Abnormalities in blood counts should not raise suspicions solely of hematological diseases. Such an approach can significantly delay the diagnostic process, thereby reducing the patient’s chances for longer survival and better treatment outcomes. The changes we have described should raise oncological vigilance toward a potential proliferative process occurring in the organs, as seen in this case. Therefore, the most important takeaway is that imaging tests with high sensitivity in detecting neoplastic foci should be ordered early in the diagnostic process.

In conclusion, the case of bone marrow metastatic angiosarcoma originating from the spleen provides valuable insight into the diverse clinical manifestations and complex biological behavior of angiosarcoma. It underscores the imperative for precise diagnosis and treatment of this malignancy.

## Figures and Tables

**Table 1:** Selected case reports of splenic angiosarcoma with associated abdominal symptoms, metastases, treatment and outcome.

Citation	Age, Sex	Abdominal Symptoms	Metastases	Treatment	Follow-Up	Outcome
Levy et al. [5]	83, F	Pain	Bone	Splenectomy + doxorubicin	1 month	Died
Frontario et al. [6]	56, F	Pain	Bone	Elective splenectomy, adriamycin and ifosfamide with mesa, paclitaxel	More than 12 months from initial diagnosis	Alive
Gorzelak-Pabis et al. [7]	78, M	Pain	Liver, infiltration in gastric and peritoneal region	Splenectomy	2 months	Died
Plantiga et al. [8]	67, F	No	Liver, bone marrow	Not reported	7 days	Died
Hasan Sözel et al. [9]	65, M	Pain	Liver	Splenectomy + systemic chemotherapy	Not reported	Not reported
Kimura et al. [10]	77, F	No	Liver	Splenectomy	1.5 months	Died
Deng et al. [11]	64, M	Vomiting	No	Splenectomy + adjuvant chemotherapy	9 months	Not reported
Duan et al. [12]	65, M	Pain	Liver	Splenectomy	6 months	Died
Zhao et al. [13]	44, M	Distention	Liver	Not started	8 h	Died
Batouli et al. [14]	45, F	Not reported	Liver	Splenectomy + chemotherapy	5 months	Died
Fang Chen et al. [15]	72, F	Pain	Liver	No data	4 weeks	Died
Wheelwright et al. [16]	50, F	Pain	No	Splenectomy + PLD and ifosfamide	4 years, then no data	Alive
Wheelwright et al. [16]	72, M	No	Spine	Splenectomy + PLD and ifosfamide	5 years, then no data	Alive
Fiorentino et al. [17]	80, F	Pain	Liver, peritoneum	Splenectomy + Paclitaxel, β-AR antagonists	6 months	Died
Schmidt de Azevedo et al. [18]	57, F	Pain	Spine	Splenectomy + Paclitaxel + Pazopanib	More than 3 years	Died
Filip Kohutek et al. [19]	65, F	Pain	Multiple in bones, liver, axial skeleton and lung	Splenectomy + Doxorubicin + Radiotherapy	13 years	Alive
Rui-Qi Zou et al. [20]	67, F	Enlarged spleen	Liver	Hepato-splenectomy	3 months	No data
Kamran S Hamid et al. [21]	70, F	Discomfort	Liver, lungs	Splenectomy + chemotherapy	8 months	No data
Barış Özcan et al. [22]	65, F	Pain and distention	Liver, bones	Splenectomy + chemotherapy	More than 5 months	No data
Soumaya Anoun et al. [23]	25, F	Splenomegaly	Bone marrow	Splenectomy	1 year	Died
Takehara et al. [24]	62, F	Left flank pain	Liver, vertebrae	Paclitaxel, doxorubicin, pazopanib, docetaxel, gemcitabine plus docetaxel, ifosfamide	23 months	Died
Shimin Hu et al. [25]	83, M	No	Bone marrow, presumably in thyroid gland	Hospice care	No data	No data
Lichen Xu et al. [26]	38, F	Pain	Liver, bone marrow	Refuse chemotherapy	3 months	Died
Wang et al. [27]	36, M	Abdominal mass localized to the left upper quadrant	Bone marrow, retroperitoneal lymph nodes, liver, lumbar spines, right kidney	Adriamycin, isophosphamide, VP-16, toxol	24 years after diagnosis	Died

**Table 2:** Complete blood count (CBC) on admission.

Parameter	CBC on Admission
WBC	1.86 × 10^3^/μL
RBC	3.15 × 10^6^/μL
Hemoglobin	9.5 g/dL
Hematocrite	27.1%
MCV	86.0 fL
MCH	30.2 pg
MCHC	35.1 g/dL
PLT	24 k/mcL
RDW-SD	51.6 fL
RDW-CV	17.8%
NEU	13.20 × 10^3^/μL
LYM	3.80 × 10^3^/μL
MON	1.34 × 10^3^/μL
EOS	0.21 × 10^3^/μL
BAS	0.03 × 10^3^/μL
NRBC	2.900 × 10^3^/μL
IG	4.00 × 10^3^/μL

## Data Availability

The authors confirm that the data supporting the findings of this study are available within the article.
